# Brg1 Supports B Cell Proliferation and Germinal Center Formation Through Enhancer Activation

**DOI:** 10.3389/fimmu.2021.705848

**Published:** 2021-09-01

**Authors:** Dominik Schmiedel, Hadas Hezroni, Amit Hamburg, Ziv Shulman

**Affiliations:** Department of Immunology, Weizmann Institute of Science, Rehovot, Israel

**Keywords:** Brg1, BAF, SWI/SNF, chromatin remodeling, enhancer activation, B cells, germinal center, antibody-formation

## Abstract

Activation and differentiation of B cells depend on extensive rewiring of gene expression networks through changes in chromatin structure and accessibility. The chromatin remodeling complex BAF with its catalytic subunit Brg1 was previously identified as an essential regulator of early B cell development, however, how Brg1 orchestrates gene expression during mature B cell activation is less clear. Here, we find that Brg1 is required for B cell proliferation and germinal center formation through selective interactions with enhancers. Brg1 recruitment to enhancers following B cell activation was associated with increased chromatin accessibility and transcriptional activation of their coupled promoters, thereby regulating the expression of cell cycle-associated genes. Accordingly, Brg1-deficient B cells were unable to mount germinal center reactions and support the formation of class-switched plasma cells. Our findings show that changes in B cell transcriptomes that support B cell proliferation and GC formation depend on enhancer activation by Brg1. Thus, the BAF complex plays a critical role during the onset of the humoral immune response.

## Introduction

Long-lasting protection from harmful pathogens depends on the efficient generation of high-affinity antibodies (1). In response to vaccination or pathogen invasion, naive B cells that reside in follicles of secondary lymphoid organs interact with cognate antigens through their B cell receptors (BCRs) and present antigen-derived peptides on surface MHC class II to cognate T helper cells (2). At this stage, cognate T cells select B cells for the generation of short-lived plasmablasts or for differentiation into germinal center (GC) cells (3). GCs are microanatomical sites in which activated B cells rapidly divide and introduce somatic hypermutations (SHM) into their immunoglobulin genes followed by affinity-based-selection (4). The major function of the GC reaction is to produce memory and antibody-forming cells that depart the lymphoid organs and provide long-lasting immunity (5). The process of B cell activation and differentiation into GC, memory, or plasma cells (PCs) highly depends on changes in gene expression that is regulated at the transcriptional and post-transcriptional levels (6, 7). Whereas the specific transcription factors that drive B cell activation and differentiation were previously described, less is known about the regulation of gene expression through changes in chromatin accessibility and structure. /B cell state transitions are guided by well-defined transcription factors such as BCL-6, BLIMP1, and PAX5 (8). However, in order to access their target sites, the chromatin structure must assume an accessible state, a process that is controlled by chromatin remodeling complexes (CRCs). The required establishment of nucleosome-depleted regions (NDRs) by shifting or evicting nucleosomes is one of the main functions of CRCs (9). NDRs are not only critical for transcription factor binding, but also for binding of cohesin and mediator complexes which create three-dimensional DNA structures, like the formation of loops between promoter regions and enhancers. These loop formations are a prerequisite for lineage-specific gene transcription as they bring transcription factors that bind distal enhancers in close proximity to the promoter of its target genes and are guided by the presence of histone modifications such as H3K4me1 or H3K27ac (10).

The BAF (BRG1/BRM-associated factor) CRC, also known as SWI/SNF complex, is particularly well-known for its capacity to form NDRs. This complex consists of up to 15 subunits and the incorporation of different subunits into it allows cell-specific functions (11, 12). The SWI/SNF complex possesses several subunits with DNA and histone recognition domains that can guide complex localization not only by DNA sequence recognition but predominantly by DNA architecture and pre-existing histone modifications (13, 14). The core of the complex is the ATPase subunit, which engages with the nucleosome-bound DNA and hydrolyzes ATP to induce a conformational change of the complex and enforces the nucleosome repositioning. Each complex possesses a single ATPase unit, which can be either Brm or Brg1 (encoded by *Smarca2* and *Smarca4*, respectively). Brg1 has a critical role in many physiological settings such as in maintaining pluripotency in stem cells (15, 16), neural development (17), and heart muscle development (18). On top, Brg1 and other complex subunits were found to be frequently mutated in diverse malignancies (13, 19), highlighting their essential role in maintaining transcriptional stability in a variety of tissues. Thus, Brg1 acts as both a tumor suppressor (18, 20) and a tumor driver (14, 21).

In the context of B cells, gene expression regulation by Brg1 was primarily studied in the process of B development in the bone marrow (BM) wherein the SWI/SNF complex plays a critical role (22). In developing B cells in the BM, Brg1 promotes fate decisions of lymphoid progenitor cells and has critical functions in pro- and pre-B cell stages. Specifically, Brg1 is required for the function of lineage-specific transcription factors like Ikaros and Pax5 through enabling access to enhancers, such as the Myc super-enhancer (23, 24). EBF1, a pioneering transcription factor, was shown to recruit Brg1 and promote phase separation and chromatin accessibility (25). Also, the contraction of the BCR heavy chain locus during the VDJ-recombination requires Brg1 functions (24).

Unlike in B cell development, understanding of the functions and mechanism of Brg1 in mature B cells remains less clear. A role for Brg1 in class-switch recombination and proliferation was previously suggested in a B cell line (26) and Srg1, a subunit of the SWI/SNF complex, was found to be required for GC formation (22). In contrast, a study that characterized changes in chromatin structure in B cells upon activation found only minor changes in Brg1 genomic occupancy, however, effects on gene expression were not examined (27). Thus, how Brg1 controls mature B cell activation and functions through chromatin modulation is not entirely solved.

Here, we find that Brg1 is critical for establishing the gene expression profile of activated B cells, by promoting chromatin accessibility at enhancers. This process allows Brg1 to activate the transcription of genes essential for cell cycle progression and ultimately GC formation. Thus, our findings define Brg1 as a key chromatin regulator that supports activation-induced transcription factors activity during the establishment of antibody-mediated immunity.

## Materials and Methods

### Mice

CD23^Cre^, γ1^Cre^ and Brg1^fl/fl^ (28) mice were purchased from the Jackson Laboratories. C57BL/6 wild-type mice were purchased from Envigo. All mice were bred and housed in specific-pathogen-free conditions. Littermate controls used as control animals were Brg1^+/+^, CD23^+/+^ or γ1^+/+^. All experiments were approved by the Weizmann Institute Animal Care and Use Committee (IACUC) with animals aged between 7-14 weeks. For immunizations, NP-KLH was emulsified in complete Freud’s adjuvant (CFA). Per mouse, 50 µl were subcutaneously injected close to the base of the tail. Animals were anesthetized with a mixture of ketamine, xylazine and acepromazine prior to the injection. 7 days after the immunization, inguinal lymph nodes were harvested and analyzed in flow cytometry.

### Method Details

#### Enzyme-Linked Immunosorbent Assay

Serum was collected from unimmunized mice, by drawing blood into a heparin-coated microcapillary. Haematocrit was removed by centrifugation (800xg, 15 minutes, 4°C) and supernatant, which represents the serum fraction, was collected. Serum was then diluted 1: 40 000 in PBS, and IgM, IgG1, IgG3 antibodies were detected by ELISA using anti-mouse IgM-, anti-mouse IgG1-, or anti-mouse IgG3 horseradish peroxidase (HRP), using 1-step-TMB-ELISA substrate and stop solution. The optical density at 450 nm (OD450nm) was measured with a microplate reader (Tecan).

#### *In Vitro* Activation of B Cells

Splenic B cells were isolated by forcing the tissue through a filter mesh into PBS containing 2% fetal calf serum and 1 mM EDTA. The cell mixture was then subjected to erythrocyte lysis by ACK buffer for 10 min, then washed twice with PBS. B cells were isolated with the Ly-48 B cell isolation kit according to the manufacturer’s instructions. Cells were cultivated in RPMI1640 medium including 25 mM HEPES, supplemented with 10% fetal calf serum (FCS), L-glutamine, pyruvate, non-essential amino acids, β-mercapto-ethanol, and activated with 10 µg/mL LPS and 20 ng/mL IL-4 for 72 or 96 hours. Cells were seeded in a density of 1 million per mL. If proliferation was examined, cells were stained with Cell Trace Violet dye according to the manufacturer’s instructions prior to the activation.

#### Flow Cytometry

Spleens, lymph nodes and Peyer’s patches were harvested and forced through a filter mesh into PBS containing 2% fetal calf serum and 1 mM EDTA. BM was collected from the hind limbs. Splenic single cell suspensions and BM samples were treated with ACK buffer in order to lyse erythrocytes. *In vitro* activated cells were mixed well, harvested and washed one with PBS.

On ice, single cell suspensions were subjected to 1 µg/ml anti-CD16/32 for 5 min in order to block nonspecific binding to FC receptors, then fluorescently labeled antibodies were added for another 25 min. Cells were gated as live and single according to their properties in FSC and SSC, then defined as follows: lymph node/Peyer’s patch/spleen: B cells: B220+ CD138-, PCs: CD138+, germinal center B cells: B220+ CD38- FAS+; BM: B cells: B220+ CD138-, PCs: CD138+; *in vitro* activation - B220+; median fluorescence intensities plotted in all diagrams were either tested with student’s t-test (2 groups) or one-way ANOVA with *post hoc* Tukey’s multiple comparisons test. Statistics were calculated in Graph Pad Prism 8. Following p values are represented by the asterisks’: p<0.05 = *; p<0.01 = **; p<0.001 = ***; p<0.0001 = ****, p>0.05 = ns (not significant). All antibodies were purchased from Biolegend.

For intracellular staining, cells were fixed after surface staining and fixed and permeabilized with the BD Cytofix/Cytoperm Kit according to the manufacturer’s instructions and then stained with anti-Blimp-1-Alexa Fluor 647 and anti-IRF4-Alexa Fluor 488 (Biolegend).

#### Immunofluorescence

Immunized inguinal lymph nodes were excised, washed in PBS, and fixed with 4% paraformaldehyde for 16 hours at 4°C. The tissues were then subjected to 30% sucrose overnight, and then fresh sucrose solution for 4 more hours before being embedded in OCT freezing solution (Tissue-Tek). 10-mm sections were cut and dehydrated in acetone prior to freezing. Sections were rehydrated in PBS and incubated with 1% SDS in PBS for 5 min, then blocked in PBS with 0.05% Tween-20 and 3% BSA for at least one hour. Slides were probed with 1:100 rabbit-anti-mouse Brg1 (clone H-88) and 1:100 anti-mouse CD35-Biotin overnight, then washed three times and then stained with anti-rabbit Alexa Fluor-488, Alexa Fluor-647 conjugated streptavidin, anti-mouse IgD - PE (each 1:200) and in 1% BSA in PBST, incubated once more overnight. Slides were washed in PBS and nuclei were counterstained for 5 min with Hoechst 33342 (1:4000) (Thermo Fisher Scientific). Sections were mounted with a mounting medium (Sigma-Aldrich) and imaged with a Zeiss LSM 880 confocal microscope.

#### Quantitative PCR Analysis and RNA Sequencing Sample Preparation

Activated splenic B cells were harvested after 72 hours activation using LPS and IL-4. Polyadenylated RNA was isolated using Dynabeads mRNA Purification Kit (ThermoFisher) according to the manufacturer’s instructions. For qPCR, total RNA was subjected to cDNA synthesis using qScript synthesis kit (Quantabio). qPCR mix was prepared using SYBR green (Roche) with primers specific for Brg1 (fw: CAAAGACAAGCATATCCTAGCCA; rv: CACGTAGTGTGTGTTAAGGACC) or Brm (fw: AGCCAGATGAGTGACCTGC: rv: TGCTTGGCATCCTTTTCGGAA). Relative transcript expression was calculated using the ddCt method and all transcripts were normalized to Actin B. For RNA sequencing, libraries were generated for bulk sequencing using the MARSseq protocol as previously described.

#### RNAseq Data Analysis

Alignment and differential expression analysis were performed using the UTAP pipeline (29): Reads were trimmed using Cutadapt and mapped to the mm10 genome assembly using STAR (30) v2.4.2a with default parameters. The pipeline quantifies the genes annotated in Gencode, extended by 1,000 bases toward the 5′ edge and 100 bases in the 3′ direction. Counting of sequenced reads was done using htseq-count (31). Genes having a minimum of five UMI-corrected reads in at least one sample were included in the analysis. Normalization of the counts and differential expression analysis was performed using DESeq2 (32). Genes were considered differentially expressed if they had a FC ≥ 2 or ≤ 2, and padj < 0.05 in Brg1^fl/fl^ cells compared to Brg1^fl/+^ and littermate controls. Heatmap of differentially expressed genes was generated with the pheatmap R package.

#### Western Blot

*In vitro* cells were suspended with ice-cold RIPA buffer (10mM Tris-HCl, pH 8.0, 140mM NaCl, 1mM EDTA, 1% Triton X-100, 0.1% Sodium Deoxycholate, 0.1% SDS). Extracts were centrifuged (15,000 xg for 15 min at 4°C), and supernatants were boiled for 5 min in SDS sample buffer. Equal amounts of protein (30 μg/well) were loaded onto an 8% SDS-PAGE. After electrophoretic separation, proteins were transferred to a nitrocellulose membrane blocked for 1 h (5% nonfat dry milk and 0.5% Tween in Tris-buffered saline), and incubated overnight at 4°C with primary antibodies. After washing, membranes were then incubated with secondary anti-mouse or anti-rabbit horseradish peroxidase-conjugated Abs (Jackson ImmunoResearch Laboratories, West Grove, PA, USA) for 1 h and exposed to ECL reagent (Thermo Fisher Scientific, Waltham, MA, USA).

Antibodies that were used: rabbit anti-human Brg1 (1:500; Cell Signaling Technology, Danvers, Massachusetts, USA), mouse anti-human beta-Actin (1:1000; Thermo Fisher Scientific, Waltham, MA, USA).

#### ATAC-seq Library Preparation

ATAC-seq was performed as previously described (33) with minor adjustment. Cells were collected after 4 days of *in vitro* activation as mentioned above. 50,000 cells were centrifuged at 400 xg for 3 min, followed by a wash using 50 μL of cold PBS and centrifugation at 400 xg for 3 min. Cells were lysed using a cold lysis buffer (10 mM Tris-HCl, pH 7.4, 10 mM NaCl, 3 mM MgCl2 and 0.1% IGEPAL CA-630). Immediately after lysis, nuclei were spun at 400 xg for 10 min using a refrigerated centrifuge. Next, the pellet was resuspended in the transposase reaction mix (25 μL 2 × TD buffer, 2.5 μL transposase (Illumina) and 22.5 μL nuclease-free water). The transposition reaction was carried out for 30 min at 37°C and immediately put on ice. Directly afterward, the sample was purified using a QIAGEN MinElute kit. Following purification, the library fragments were amplified using custom Nextera PCR primers 1 and 2 for a total of 12 cycles. Following PCR amplification, the libraries were purified using a QIAGEN MinElute kit and sequenced with paired-end sequencing using NovaSeq 6000.

#### ATAC-seq Data Analysis

Reads were aligned to the mm10 genome assembly using Bowtie2 (34). Normalized read coverage files were computed by deepTools (35). Peaks were called using MACS2 (36) and annotated using HOMER (37). Peaks from the eight analyzed datasets were combined. Read coverage in the peaks was computed using bigWigAverageOver bed UCSC utility (38). Differential accessibility was computed with DESeq2 (32).

#### ChIP-seq Data Analysis

A previously published Brg1 ChIP-seq dataset [GSE82144 (27)] was used for the identification of Brg1 binding sites. Reads were aligned to the mm10 genome assembly with Bowtie2 (34). Peak calling was done using MACS2 (36), and peaks were annotated using HOMER (37).

#### Functional Annotation of Brg1 Target Genes

Enriched GO terms within down- or upregulated Brg1 target genes were identified using GOrilla (39). Enriched GO terms were then summarized by REViGO (40). Gene Set Enrichment Analysis (GSEA) was performed using GSEA 4.1 (41). Gene names were converted to human gene symbols, and software was run with default parameters, using the “hallmark” signatures from the MSigDB database (42). Enrichment for transcription factors binding to promoters within down- or upregulated Brg1 targets was done using Enrichr (42, 43), with the Chea database (44).

#### Analysis of Brg1 Bound Enhancers

Heatmaps representing ChIP-seq signals for Brg1, H3K4me1 and H3K27ac were generated using deepTools (35). Bigwig files used heatmaps were downloaded from GEO (GSE82144). Genomic coordinates of FAIRE-seq and STARR-seq peaks, and of promoter-enhancer interactions were downloaded from GEO (GSE121753) (45). Overlap between Brg1 bound regions and enhancer regions was calculated using bedtools intersect. Signals of Brg1 over different genomic regions were generated using deepTools (35). Mean Brg1 coverage over different genomic regions was calculated using bigWigAverageOverBed.

## Results

### Brg1 Controls Transcription Regulation in Activated B Cells

**Figure 1:**
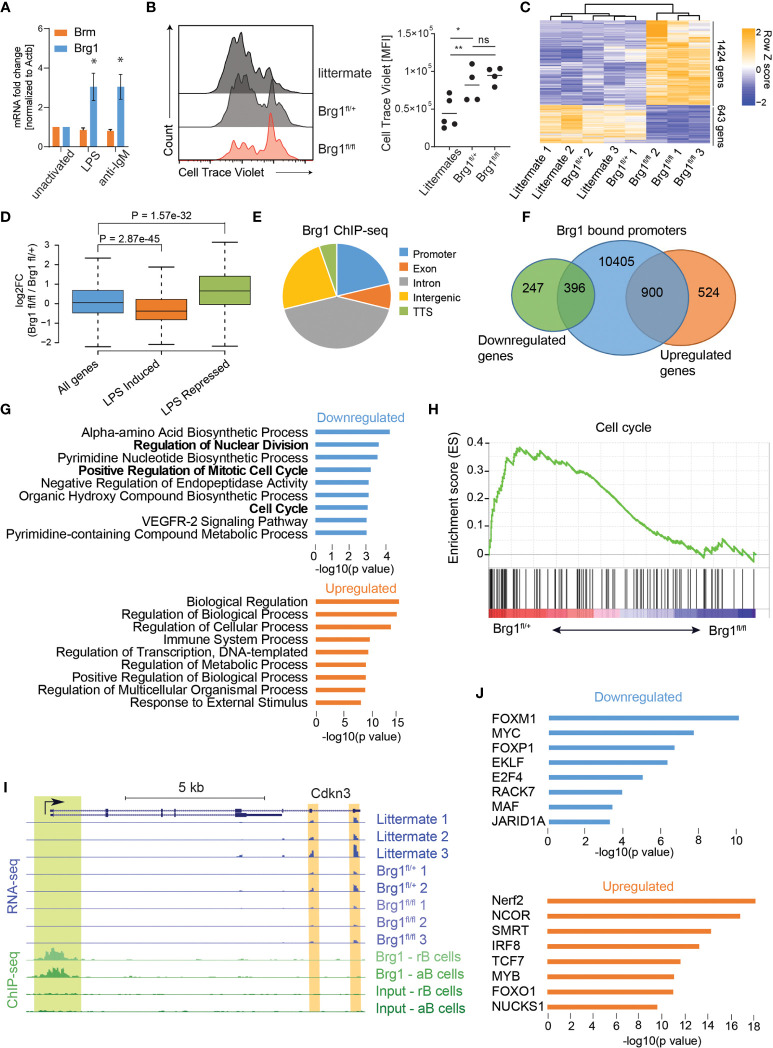
Brg1 regulates the expression of cell cycle genes in activated B cells **(A)** qRT-PCR analysis of *Smarca2* and *Smarca4* mRNA levels of B cells following treatment with anti-IgM or LPS for 2 days, n = 3 mice, * p<0.05 in a one-sample t-test compared to unactivated cells. **(B)** Flow cytometric analysis of splenic B cell proliferation following 72 hours of LPS and IL-4 activation. MFI of CellTrace Violet cell tracker dye is shown and quantified. Each dot represents one mouse. Statistics were calculated with one-way ANOVA with *post hoc* Tukey’s multiple comparisons test. p<0.05 = *; p<0.01 = **; p>0.05 = ns (not significant). **(C)** Heat map representation of clustering analysis of differentially expressed genes. All genes with padj<0.05 and fold change of at least 2 are shown. **(D)** Boxplots indicating the median, quartiles, and 5th and 95th percentiles of changes in expression levels of CD23-Cre Brg1^fl/fl^ compared to Brg1^fl/+^ B cells in genes induced or repressed following LPS activation compared to total genes. P value was calculated by a two-sided Wilcoxon rank sum test. **(E)** Distribution of 70,333 Brg1-binding sites across genomic regions in LPS activated B cells. TTS: transcription termination site. **(F)** Venn diagram showing overlap between 11,701 Brg1 bound promoters, 1,424 genes upregulated in Brg1^fl/fl^ compared to Brg1^fl/+^ cells, and 643 genes downregulated in Brg1^fl/fl^ compared to Brg1^fl/+^ cells. **(G)** GO terms which were enriched in genes whose promoters were bound by Brg1 and were down- or upregulated in CD23-Cre Brg1^fl/fl^ compared to Brg1^fl/+^ B cells. **(H)** Gene set enrichment plot for cell cycle genes from the hallmark gene sets. **(I)** UCSC genome browser tracks showing the locus of Cdkn3 (padj < 0.05). Tracks show the expression levels, measured by RNA-seq, in activated B cells and ChIP-seq signal of Brg1 in resting and activated B cells, compared to input controls. Green highlight: binding of Brg1 in the promoter region. Orange highlights: 3’ end exons covered by RNA-seq. **(J)** Transcription factors enriched for binding the promoters of genes which were bound by Brg1 and were down- or upregulated in Brg1^fl/fl^ compared to Brg1^fl/+^ cells.

In order to examine how Brg1 controls the activation of mature B cells, we examined changes in gene expression patterns in B cells stimulated through TLR4 or the BCR. In this context, LPS stimulation or IgM crosslinking of B cells induced expression of Brg1 but not of Brm (**Figure 1A**). In order to examine the role of Brg1 in B cell immune responses, we crossed Brg1^fl/fl^ mice to a transgenic mouse strain that expresses Cre specifically in mature B cells (CD23-Cre). In B cells derived from these mice, a significant reduction in Brg1 protein levels was observed (**Figure S1A**). As opposed to commonly used B cell-specific mouse models like CD19-Cre, deletion of genes in this mouse strain takes place during their final differentiation in the spleen after their departure from the BM (46). LPS-stimulated B cells derived from littermate control mice showed effective proliferation, whereas B cells lacking one *Smarca4* allele were moderately impaired, and the proliferation of B cells lacking both *Smarca4* alleles was significantly reduced (**Figure 1B**). Yet, Brg1-deficient B cells were able to respond to LPS stimulation as they showed CD86 upregulation 18 hours after activation (**Figure S1B**) and no reduction in cellular viability was observed (**Figure S1C**). To identify the genes and pathways affected by the loss of Brg1, LPS-activated B cells were subjected to RNA-seq. We found that 643 genes were downregulated and 1424 genes were upregulated by at least twofold in Brg1-deficient B cells compared to littermate controls (**Figure 1C**). Comparing our data to a dataset of gene expression following eight hours of LPS activation of splenic B cells (47), we found that genes that were induced following LPS activation were significantly downregulated in Brg1-deficient B cells, while genes that were repressed following LPS activation were upregulated in Brg1-deficient cells (**Figure 1D**). To characterize how genome-wide occupancy of Brg1 affects gene expression, we first examined a pre-existing dataset of Brg1 ChIP-seq of resting and LPS activated splenic B cells (27). Brg1 occupancy was abundant, with over 50,000 peaks in resting B cells and over 70,000 peaks in activated B cells. Similar to previous findings in pro-B cells (24), Brg1 peaks in mature activated B cells were localized mainly to introns and intergenic regions, while less than a third of the peaks were localized to gene promoters (**Figure 1E**). Analyzing the promoter-bound genes based on their transcriptional response to Brg1 loss, we found that 900 upregulated genes and 396 downregulated genes had Brg1 peaks in their promoters (**Figure 1F**), suggesting their transcription may be directly regulated by this chromatin remodeler. GO analysis and gene set enrichment analysis (GSEA) showed that downregulated Brg1 targets were enriched for cell cycle progression and anabolic process (**Figures 1G, H**), in agreement with the weak proliferation observed in stimulated Brg1-deficient B cells *in vitro*. Cdkn3 and E2f8, two regulators of cell cycle progression, were downregulated in Brg1-deficient B cells and showed Brg1 peaks in their promoters (**Figure 1** and **Figure S1D**). These findings demonstrate that Brg1-deficient cells fail to acquire the proper transcriptional program that promotes B cell activation in response to LPS. In addition, GSEA analysis showed downregulation of Myc and E2f target genes which are essential for proper B cell proliferation (**Figure S1E**). To examine whether Myc and E2f transcription factors directly regulate the expression of downregulated Brg1 targets, we analyzed the promoters of these genes for binding by transcription factors using the Chea database, which contains ChIP-seq and ChIP-chip experiments for 199 transcription factors (44). We found that promoters of downregulated Brg1 targets were enriched for binding by Myc and E2f4, in addition to other transcription factors that play a role in cell cycle progression and cell proliferation including Foxm1 and Foxp1 (**Figure 1J**). Indeed, *E2f4* expression was reduced in Brg1-deficient B cells (**Figure S1F**). In contrast, despite the fact that MYC targets were less expressed in Brg1-deficient B cells, and that many downregulated Brg1 targets were regulated by MYC, the expression of *Myc* gene was not significantly reduced in Brg1-deficient cells compared to control cells (**Figure S1F**). These data suggest that in activated B cells, Brg1 doesn’t control Myc expression directly. Upregulated Brg1 target genes were enriched for GO terms related to immune system processes and transcriptional regulation, including Bach2 transcriptional regulator (**Figure 1G** and **Figure S1C**). The promoters of these genes were enriched for binding by NCOR and SMRT (**Figure 1J**), two transcriptional repressors which are known to act in Brg1 containing complexes (48) that play important roles in transcription regulation in B cells (49, 50). Taken together, these data suggest that Brg1 plays important roles in transcriptional regulation in activated B cells, both through activation of genes required for cell proliferation and in repression of genes that are downregulated in response to LPS activation.

### Brg1 Promotes Enhancer Chromatin Accessibility in Activated B Cells

**Figure 2:**
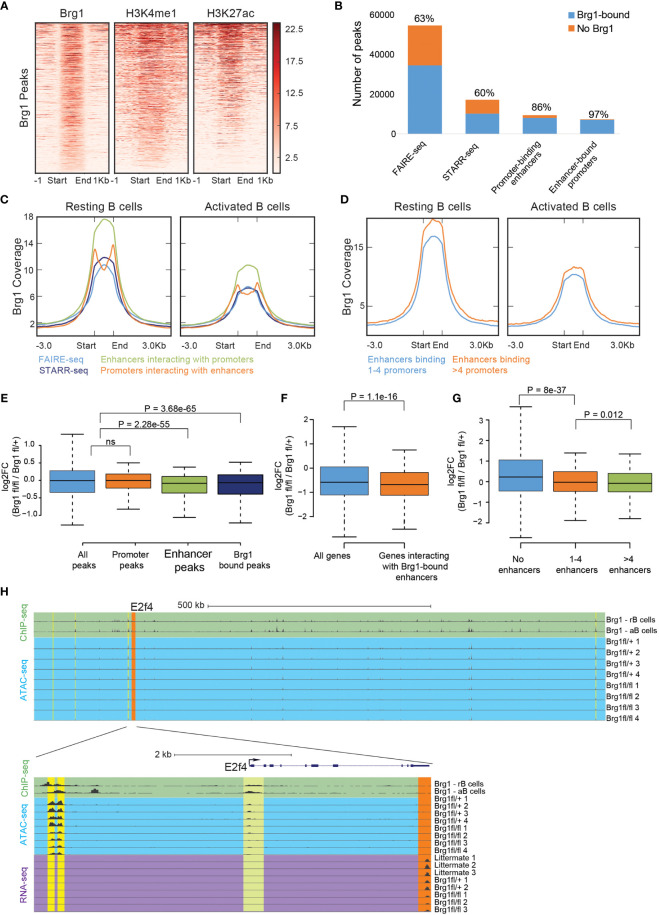
Brg1 recruitment to multiple enhancers is associated with transcriptional activation of their coupled promoters. **(A)** Density of ChIP-seq reads for Brg1, H3K4me1, and H3K27ac in activated B cells. Plots show ±1 kb around the midpoint of each Brg1-enriched region ranked according to Brg1 density. **(B)** The numbers of FAIRE-seq peaks, STARR-seq peaks, promoter-binding enhancers, and enhancer-bound promoters, and the fraction of each group that overlaps Brg1 bound genomic regions. **(C)** Averaged tag densities of Brg1 are plotted across all FAIRE–seq peaks, STARR-seq peaks, promoter-binding enhancers, and enhancer-bound promoters, in resting and activated B cells. **(D)** Averaged tag densities of Brg1 are plotted across enhancers binding 1-4 promoters compared to enhancers binding >4 promoters, in resting and activated B cells. **(E)** Boxplots indicating the median, quartiles, and 5th and 95th percentiles of changes in ratios of ATAC-seq signal for the indicated group of peaks, comparing CD23-Cre Brg1^fl/fl^ cells to Brg1^fl/+^ B cells; p>0.05 = ns (not significant). **(F)** Boxplots indicating the median, quartiles, and 5th and 95th percentiles of changes in expression levels of Brg1^fl/fl^ cells compared to Brg1^fl/+^ cells in genes whose promoters interact with Brg1 bound enhancers compared to all genes. P-value was calculated by the two-sided Wilcoxon rank-sum test. **(G)** Boxplots indicating the median, quartiles, and 5th and 95th percentiles of changes in expression levels of CD23-Cre Brg1^fl/fl^ cells compared to Brg1^fl/+^ cells in genes whose promoters interact with 1-4 enhancers or with more than 4 enhancers, compared to genes whose promoters were not found to interact with active enhancers. P values were calculated by a two-sided Wilcoxon rank-sum test. **(H)** Top: UCSC genome browser tracks showing the area around the E2f4 gene, which was significantly downregulated in Brg1^fl/fl^ cells (padj < 0.05). Tracks show the ChIP-seq signal of Brg1 in resting and activated B cells and ATAC-seq coverage in activated B cells. Orange highlight: E2f4 gene. Yellow highlights: enhancers which were found to interact with the promoter of E2f4. Bottom: zoom-in to the E2f4 locus, showing ChIP-seq signal of Brg1 in resting and activated B cells, ATAC-seq coverage and expression levels measured by RNA-seq. Green highlight: binding of Brg1 in the promoter region of E2f4. Orange highlight: 3’ end exons covered by RNA-seq. Yellow highlights: enhancers which were found to interact with the promoter of E2f4. rB cells, resting B cells. aB cells, activated B cells.

Brg1 was previously reported to activate cell-type-specific enhancers by facilitating the depletion of nucleosomes in pro-B cells (24) and in mesoderm lineage commitment (51). In activated B cells, Brg1 bound regions were marked by H3K4me1 and H3K27ac, two histone modifications that typically mark enhancers (**Figure 2A**). In order to study whether Brg1 affects gene expression through enhancer activation, we utilized previously published datasets, including enhancer regions that were mapped by FAIRE-seq and tested for functionality using STARR-seq, and enhancer-promoter interactions mapped by Hi-C (45). FAIRE-seq peaks define regions of open and accessible chromatin. STARR-seq peaks define regions that are both accessible and were shown to act as functional enhancers using a high-throughput screen. Open chromatin regions that were not validated as functional enhancers might represent poised enhancers with the potential to rapidly become activated under specific cellular contexts (45). We found that Brg1 was bound to most poised and active enhancers (represented by FAIRE-seq and STARR-seq peaks, respectively), and to even larger fractions of enhancer-promoter pairs (as detected by Hi-C analysis) (**Figure 2B**). Brg1 signal was stronger in resting B cells compared to their activated counterparts, and stronger in STARR-seq validated enhancers compared to all FARE-seq regions (**Figures 2C**, **S1G**). We also found that the Brg1 signal was stronger in enhancers that interact with multiple promoters (**Figure 2D** and **Figure S1H**), which were reported to have increased chromatin accessibility, suggesting that Brg1 preferentially interacts with enhancers that show higher activity. In order to examine the effect of Brg1 deficiency on the chromatin landscape we used ATAC-seq to map accessible chromatin regions. We compared B cell-derived from CD23-Cre Brg1^fl/fl^ and CD23-Cre Brg1^fl/+^ that showed similar gene expression patterns as Brg1-sufficient littermates in RNA-seq analysis (**Figure 1C**). We obtained 16,464 peaks representing open chromatin regions, and for each peak computed the average ratio of read coverage between Brg1-deficient and control B cells. 752 peaks showed significantly reduced chromatin accessibility in Brg1-deficient B cells, and 340 of those peaks overlapped with activated B cells enhancers (**Figure S1I**). 322 peaks showed a significant increase in chromatin accessibility in Brg1-deficient B cells, and only 20 of these peaks overlapped enhancer regions (**Figure S1I**). Nearly all of the observed peaks with reduced chromatin accessibility were also bound by Brg1 according to the ChIP-seq data, while only a third of the peaks that showed increased accessibility was bound by Brg1 (**Figure S1J**). While peaks overlapping promoters were not affected by the loss of Brg1, peaks overlapping enhancer regions, as well as peaks overlapping Brg1 binding sites, had significantly reduced chromatin accessibility in Brg1-deficient B cells (**Figure 2E**). Taken together, these data suggest that Brg1 preferentially binds enhancers in activated B cells and promotes chromatin accessibility.

We next examined specifically the expression levels of genes whose promoters interact with enhancers bound by Brg1 and found that these genes were significantly less expressed in Brg1-deficient cells (**Figure 2F**). We also found that the expression of genes that were bound by more than four enhancers was downregulated to a larger extent compared to genes bound by up to four enhancers (**Figure 2G**). Importantly, genes bound by multiple enhancers were reported to have enhanced levels of nascent transcripts compared to genes bound by single enhancers (45). An example of a gene whose promoter interacts with multiple Brg1 bound enhancers is E2f4, a transcription factor regulating cell cycle progression whose expression was downregulated in Brg1-deficient cells (**Figure S1F**). Hi-C analysis identified seven enhancers interacting with the promoter of E2f4, and our data show that these enhancers were bound by Brg1 and had decreased chromatin accessibility in Brg1-deficient cells (**Figure 2H**). These data suggest that Brg1 recruitment to multiple enhancers promotes transcriptional activation of their coupled promoters.

### Effective Antibody-Mediated Immune Response Depends on Brg1

**Figure 3:**
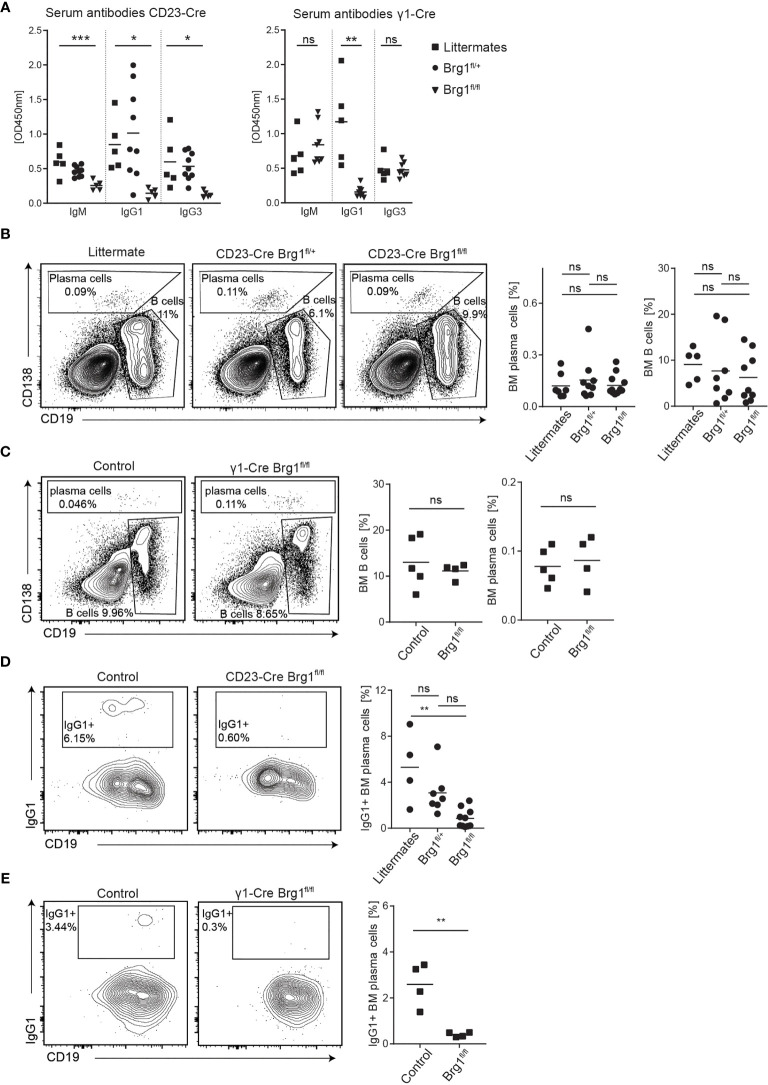
Brg1-deficiency in B cells causes a reduction in serum antibodies and class-switched plasma cells. **(A)** ELISA for IgM, IgG1, and IgG3 in the serum of unimmunized CD23-Cre and γ1-Cre littermate, Brg1^fl/+^ and Brg1^fl/fl^ mice. One-way ANOVA with *post hoc* Tukey’s multiple comparisons test. Following p values are represented by the asterisks’: p<0.05 = *; p<0.01 = **; p<0.001 = ***; p>0.05 = ns (not significant). **(B)** Analysis of BM B cells and CD138^+^ cells in CD23-Cre mice by flow cytometry. **(C)** Analysis of BM B cells and CD138^+^ cells in γ1-Cre mice by flow cytometry. **(D)** Assessment of IgG1-class-switched BM CD138^+^ cells in CD23-Cre mice using intracellular staining. **(E)** Assessment of IgG1-class-switched BM CD138^+^ cells in γ1-Cre mice using intracellular staining.

To evaluate how Brg1 functions at the chromatin level translate to effects on B cell immune responses, we examined immunoglobulin titers in the serum of unimmunized littermate control and CD23-Cre Brg1^fl/fl^ mice. This analysis revealed a significant reduction in all of the antibody isotypes in Brg1-deficient mice (**Figures 3A**). The generation of IgG1 antibodies is primarily promoted by a T cell-dependent B cell activation response and generation of GCs (52). Therefore, we also examined antibody generation in mice in which Cre is expressed during B cell activation and CSR. For this purpose, we crossed the Brg1^fl/fl^ mice to a mouse strain that expresses Cre after B cell activation during transcription of the sterile transcript of IgG1 (γ1-Cre mice (53),). In these mice, a significant reduction in IgG1 levels was observed whereas IgM and IgG3 titers were not affected (**Figures 3A**). We conclude that an effective generation of antibodies depends on Brg1.

The majority of long-lived PCs reside in the BM, where they can survive for long periods in specific niches and continuously secrete antibodies (54). Therefore, we assessed the frequency of total and IgG1^+^ BM PCs in littermates and CD23-Cre Brg1^fl/fl^ by flow cytometry analyses (**Figure S2A**). We found that the frequency of overall PCs was not different between the groups of mice. Overall the B cell fractions did not significantly change in CD23-Cre Brg1^fl/fl^ and γ1-Cre Brg1^fl/fl^ mouse strains within the BM (**Figures 3B, C**), although a small reduction was observed in the heterozygote mice. Nonetheless, the frequency of IgG1 class-switched PCs was significantly decreased, in the CD23-Cre Brg1^fl/fl^ mice and nearly undetectable in γ1-Cre Brg1^fl/fl^ mice (**Figures 3D, E**). To examine if Brg1 directly affects the expression of genes that are associated with plasma cell differentiation, we examined the expression of Irf4 and Blimp-1 in Brg1-deficient B cells following LPS activation. The expression of both of these transcription factors was intact suggesting that direct activation of plasma cell differentiation program is not dependent on Brg1 (**Figure S2B**). Collectively, we conclude that Brg1 in B cells is indirectly essential for the generation of IgG1^+^ PCs.

### Brg1 Is Required for the Generation of Germinal Center B Cells

**Figure 4:**
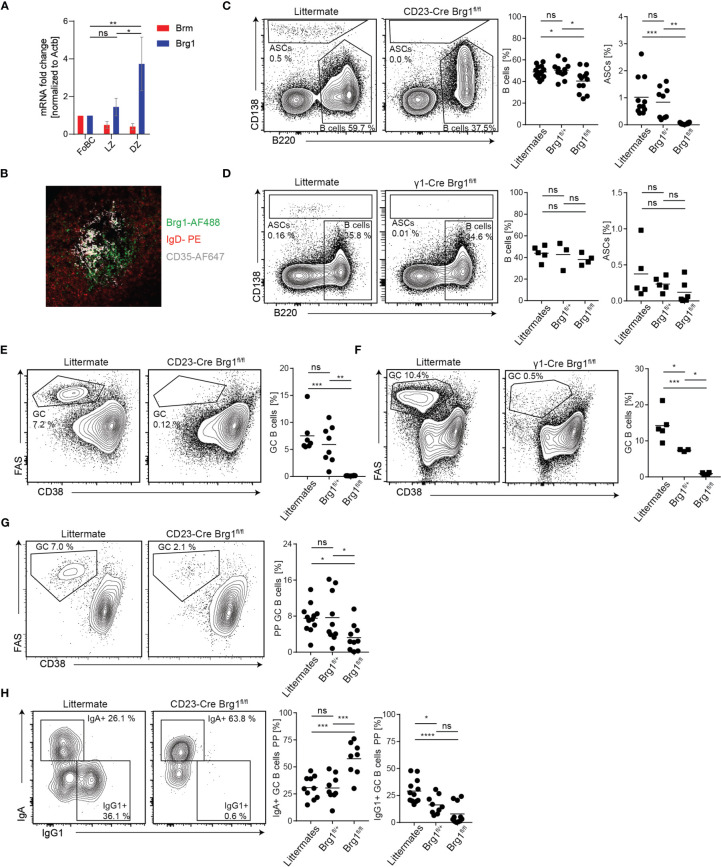
Formation of germinal centers depends on Brg1. **(A)** qRT-PCR analysis of Brm and Brg1 RNA levels relative to Actin B levels in follicular B cells (FoBC), light zone (LZ), and dark zone (DZ) B cells; n = 5 mice. One-way ANOVA with *post hoc* Tukey’s multiple comparisons test was performed, following p values for differences in Brg1 RNA levels are represented by the asterisks’: p<0.05 = *; p<0.01 = **. Changes in Brm RNA levels were not significant. **(B)** Staining for IgD-PE (naive B cells), CD35-AF647 (follicular dendritic cells in the LZ) and Brg1 (clone H-88, detected with an anti-rabbit-IgG-AF488 secondary antibody) in LN-derived from immunized mouse **(C)** Naive B cell and ASC percentages in inguinal LN determined by flow cytometry, 7d after injection of KLH in CFA. Each dot represents one mouse, n = 9 - 12 mice per group. One-way ANOVA with post hoc Tukey's multiple comparisons test was used for statistical analysis. Following p values are represented by the asterisks' in this figure: p<0.05 = *; p<0.01 = **; p<0.001 = ***; p<0.0001 = ****; p>0.05 = ns (not significant). **(D)** Naive B cell and ASC percentages in inguinal LN determined by flow cytometry 7d after injection of KLH in CFA. **(E)** The fraction of GC B cells in inguinal LN determined by flow cytometry, 7d after injection of KLH in CFA. **(F)** GC B cells in inguinal LN determined by flow cytometry in immunized γ1-Cre littermate, Brg1^fl/+^ and Brg1^fl/fl^ mice, 7d after injection of KLH in CFA. **(G)** Flow cytometric analysis of PP GCs in CD23-Cre littermate, Brg1^fl/+^ and Brg1^fl/fl^ mice. **(H)** Quantification of IgA^+^ and IgG1^+^ B cells in PP in CD23-Cre littermate, Brg1^fl/+^ and Brg1^fl/fl^ mice.

Since IgG1 antibodies typically carry SHM, the GC reaction is their primary source. Thus, we examined the possibility that the observed reduction in PC frequencies in both mouse models is a result of defects in GC formation. First, we verified that Brg1 is expressed in GC B cells. For this purpose, we purified naive, light zone (LZ) and dark zone (DZ) GC B cells from immunized mice and examined Brg1 expression by qRT-PCR. Brg1 RNA (encoded by *Smarca4*) was primarily expressed in DZ B cells (**Figure 4A**). Accordingly, we detected Brg1 expression primarily in the DZ of the GC by immunohistochemistry (**Figure 4B**). To examine if Brg1 plays a role in PC generation during a T cell-dependent immune response, CD23-Cre Brg1^fl/+^, CD23-Cre Brg1^fl/fl,^ and littermates control mice were injected with KLH in CFA and the frequency of CD138^+^ cells was examined in draining lymph nodes (LNs). Whereas a clear antibody-secreting cells (ASCs) population was detected in littermate controls and CD23-Cre Brg1^fl/+^, these cells were undetectable in LNs derived from CD23-Cre Brg1^fl/fl^ mice (**Figure 4C**, gating in **Figure S2C**). The absence of ASCs can be a result of either reduced levels of mature B cells or an inability to form GCs. The frequency of naive B cells was significantly reduced in CD23-Cre Brg1^fl/fl^ mice compared to control, however, they still hosted substantial amounts of B cells (**Figure 4C**). ASCs were not detected in the LNs of CD23-Cre Brg1^fl/fl^ and a similar trend was observed in γ1-Cre Brg1^fl/fl^ mice, although in some mice ASCs were observed in this model (**Figures 4C, D**). Most importantly, GC B cells were not detected in the LNs of immunized CD23-Cre Brg1^fl/fl^ and γ1-Cre Brg1^fl/fl^ mice (**Figures 4E, F**). We conclude that the deficiency in the generation of ASCs in response to immunization is primarily due to a severe defect in GC formation.

To further substantiate our findings, we examined the role of Brg1 in immune responses within Peyer’s patches, lymphoid organs that constantly host GCs driven by gut-derived antigens. In CD23-Cre Brg1^fl/fl^ the frequency of GC B cells in PPs was severely reduced, although in most mice, a clear GC cell population could be detected (**Figure 4G**). Remarkably, nearly all of the remaining GC B cells in PPs of Brg1^fl/fl^ mice carried an IgA BCR and very few cells were IgG1^+^ (**Figure 4H**). These findings reveal that Brg1 is important for effective formation of IgG1^+^ GC B cells in response to gut-derived antigens whereas IgA^+^ GC B cells are less dependent on Brg1. Analysis of PP GCs in γ1-Cre Brg1^fl/fl^ mice showed normal frequency of GC cells (**Figure S3A**) but nearly no IgG1^+^ B cells were detected (**Figure S3B**). In γ1-Cre mice, Cre-mediated recombination in IgA^+^ B cells in PPs is very ineffective and thus the IgA B cell compartment cannot be considered as Brg1-deficient (53). To validate a role for Brg1 in CSR, we performed *in vitro* activation of CD23-Cre splenic B cells using LPS and IL-4 and observed a significant reduction in IgG1^+^ Brg1-deficient B cells (**Figure S3C**). Collectively, we conclude that Brg1 is required for the formation of IgG1^+^ GC B cells in immune responses that are driven by gut-derived antigens within PPs.

## Discussion

In this study, we examined how Brg1 regulates gene expression through chromatin modulation in B cells. We have mapped the transcriptional response, chromatin landscape and Brg1 interactions with promoters and enhancers during B cell activation and found several hundred genes that are directly and indirectly regulated by Brg1. The majority of these genes were activation-induced genes, associated with cell cycle functions, that support B cell expansion. Specifically, we find that Brg1 promotes chromatin accessibility in enhancer regions, leading to transcriptional activation of genes that are essential for proper B cell proliferation. Accordingly, regulation of B cell activation by Brg1 is essential for GC formation and the establishment of long-lasting immunity.

What are the mechanistic details of Brg1 in regulating those genes? Our data show that Brg1 predominantly binds promoter and enhancer regions of the genes it regulates, defined by histone marks H3K27ac and H3K4me1 that occur concomitantly with Brg1 binding. These genes are then activated by diverse activating transcription factors which were previously shown to critically affect GC functions including Myc, which is essential for B cell proliferation and affinity-based selection (55, 56). In line with the binding of activating and inhibitory transcription factors at sites of Brg1 binding, we identified a subset of genes that are not induced in absence of Brg1, and also an additional subset of genes that are not sufficiently suppressed. Of note, in mature B cells, Brg1 does not play a role in the regulation of *Myc* as previously shown in developing B cells in the BM (24). Nonetheless, plenty of MYC downstream targets were downregulated in absence of Brg1, suggesting that this chromatin remodeling complex creates NRDs to enable MYC binding and function. Therefore, we define a new additional mechanism of action of the BAF complex that regulates MYC targets within the same cell lineage.

Our data show that Brg1 binds the majority of open-chromatin regions and active enhancers in activated B cells and that genes whose promoters interact with Brg1-bound enhancers are downregulated in Brg1-deficient cells. These findings support previous studies showing that Brg1 is required for enhancer activation through eviction of nucleosomes (24) and robust acetylation of chromatin (51) at enhancer regions.

We found that Brg1 recruitment to enhancers was more frequent in resting B cells than in activated B cells, suggesting Brg1 might be required for the initial activation of these enhancers, allowing the cells to acquire the proper transcriptional response to LPS activation. This is similar to H3K4me1, which marks poised enhancers and was reported to be diminished following B cell LPS activation (45). In addition, we found increased Brg1 occupancy in enhancers that interact with multiple promoters. Through these interactions, Brg1 can be involved in coordinated control over multiple genes, which can explain the strong impact of Brg1 loss on the expression of hundreds of genes. Although the possibility of a secondary effect cannot be completely excluded, the fact that we observe changes in genes whose promoters and enhancers are Brg1 targets, strongly suggests a direct effect.

Given the cell cycle-centered genetic profile related to Brg1-mediated functions, it is unsurprising that Brg1 is predominantly expressed in the DZ of the GC, as this is the primary site where GC B cells proliferate. Accordingly, both of our mouse models lack GC reactions which explain the strong defect in antibody formation. Yet, Brg1 was not essential for the generation of all bone-marrow resident PCs. Furthermore, the Brg1-deficient GC B cells observed in PPs were class-switched to IgA whereas IgG1 class-switched cells, which highly depend on T cell help, were not detected. Since IgA is less dependent on T cell help (52), these findings suggest that Brg1 might not be essential for T cell-independent responses. Thus, whereas IgG1 responses are Brg1-dependent it is most likely that the PP GCs are defective as well, further investigation to expose the role of Brg1 in CSR to IgA is required.

Collectively, our data highlight new mechanistic insights into the mode of action of Brg1 in B cell activation and raise the hypothesis that the main role of this chromatin remodeler, also in other physiological contexts, lies in the guidance of enhancer-promoter pairings. On the one hand, this would explain its striking role in the differentiation of diverse tissues, as lineage-specific transcription factors frequently bind enhancers remotely apart from their target genes. Without Brg1, the pairing of promoters and enhancers might be impaired and therefore gene transcription cannot be properly induced. On the other hand, this hypothesis also fits the seemingly contradicting roles for Brg1 in different cancer cells, both previously described as a tumor suppressor and oncogene. As Brg1 does not exert transcriptional regulation by itself but rather enables functions of other transcription factors, the impacts of a Brg1 loss depend on the transcription factor expression in a given tumor cell. Together, our findings provide an explanation for how Brg1 regulates multiple genetic programs that support cell proliferation and differentiation in healthy and pathological conditions.

## Data Availability Statement

All sequencing data have been deposited in GEO (accession number GSE180994). The data is publicly available using following link: https://www.ncbi.nlm.nih.gov/geo/query/acc.cgi?acc=GSE180994.

## Ethics Statement

The animal study was reviewed and approved by The Weizmann Institute IACUC committee.

## Author Contributions

Conceptualization, DS, HH, and ZS. Investigation, DS and AH. Formal analysis, DS and HH. Resources, DS and AH. Visualization: DS and HH, Software, HH. Data curation, DS and HH. Writing - original draft, DS and HH. Writing – review and editing, DS, HH and ZS, Supervision, project administration management, and funding acquisition, ZS. All authors contributed to the article and approved the submitted version.

## Funding

ZS is supported by the Israel Science Foundation (ISF) grant no. 1090/18 and the Morris Kahn Institute for Human Immunology. ZS is also supported by grants from The Benoziyo Endowment Fund for the Advancement of Science, The Sir Charles Clore Research Prize, Comisaroff Family Trust, Irma & Jacques Ber-Lehmsdorf Foundation, Gerald O. Mann Charitable Foundation and David M. Polen Charitable Trust. ZS is a member of the European Molecular Biology Organization (EMBO) Young Investigator Program.

## Conflict of Interest

The authors declare that the research was conducted in the absence of any commercial or financial relationships that could be construed as a potential conflict of interest.

## Publisher’s Note

All claims expressed in this article are solely those of the authors and do not necessarily represent those of their affiliated organizations, or those of the publisher, the editors and the reviewers. Any product that may be evaluated in this article, or claim that may be made by its manufacturer, is not guaranteed or endorsed by the publisher.

## Supplementary Material

Supplementary Figure 1 Brg1 is required for proper expression of cell-cycle-related genes in LPS-stimulated B cells. **(A)** Western Blot analysis for Brg1 protein levels in splenic B cells of littermate and Brg1^fl/fl^ CD23-Cre mice. B cells were activated for 4 days with LPS and IL-4. β-Actin serves as loading control. **(B, C)** Flow cytometric analysis of the live-dead marker 7-AAD as well as B cell activation marker CD86 after 18 hours of activation with LPS and IL-4. Background staining was determined from CD86-PE on B220-negative splenic cells (non-B cells). **(D)** UCSC genome browser tracks showing the loci of E2f8, which was significantly downregulated in CD23-Cre Brg1^fl/fl^ B cells, and Bach2, which was significantly upregulated in CD23-Cre Brg1^fl/fl^ cells. Tracks show the expression levels of the genes, measured by RNA-seq, in activated B cells from littermates, CD23-Cre Brg1^fl/+^, and Brg1^fl/fl^ mice, and ChIP-seq signal of Brg1 in resting and activated B cells, compared to input control. Green highlight: binding of Brg1 in the promoter regions. Orange highlights: 3’ end exons covered by RNA-seq. rB cells, resting B cells. aB cells, activated B cells. **(E)** Gene set enrichment plots for Myc targets and E2f targets from the hallmark gene sets. **(F)** Expression levels of E2f4 and Myc, measured by RNA seq, in control samples, including CD23-Cre Brg1^fl/fl^ and littermate controls. Normalization of reading counts and calculation of p value were done using Deseq2. **(G)** Boxplots indicating the median, quartiles, and 5th and 95th percentiles of mean Brg1 coverage across all FAIRE-seq and STARR-seq peaks in resting B cells. P values were calculated by a two-sided Wilcoxon rank-sum test. **(H)** Boxplots indicating the median, quartiles, and 5th and 95th percentiles of mean Brg1 coverage across all enhancers binding 1-4 promoters and enhancers binding more than 4 enhancers, in resting and activated B cells. P values were calculated by a two-sided Wilcoxon rank-sum test. **(I)** Venn diagram showing overlap between 5,841 ATAC-seq peaks overlapping activated B cells enhancers, 752 ATAC-seq peaks with significantly reduced chromatin accessibility in Brg1-deficient cells, and 322 ATAC-seq peaks with significantly increased chromatin accessibility in Brg1-deficient cells. **(J)** Venn diagram showing overlap between 13,890 Brg1 bound ATAC-seq peaks, 752 ATAC-seq peaks with significantly reduced chromatin accessibility in Brg1-deficient cells, and 322 ATAC-seq peaks with significantly increased chromatin accessibility in Brg1-deficient cells.

Supplementary Figure 2 Brg1 is not critical for the expression of Blimp-1 and IRF4 in B cells following LPS activation. **(A)** Gating strategy used for analysis of PCs in BM samples. **(B)** Analysis of IRF4 and Blimp-1 protein levels assessed by intracellular flow cytometry in CD23-Cre littermates or Brg1^fl/fl^ splenic B cells before and after 4 days activation with LPS and IL-4. Statistics were calculated with students’ t-test. **(C)** Gating strategy applied to detect GC B cells in lymph nodes and PPs.

Supplementary Figure 3 Brg1 is required for the generation of IgG1+ germinal centers B cells in Peyer’s patches. **(A)** Analysis of germinal center size in PPs of γ1-Cre control (littermate or Brg1^fl/+^) or Brg1^fl/fl^ mice analyzed by flow cytometry. Statistic s were calculated with students’ t-test. **(B)** Analysis of class-switch recombination to IgA and IgG1 in Peyer’s patches of γ1-Cre control (littermate or Brg1^fl/+^) or Brg1^fl/fl^ mice analyzed by flow cytometry. Statistics were calculated with students’ t-test. **(C)** Analysis of class-switch recombination to IgG1 in splenic B cells derived from littermate, Brg1^fl/+^ or Brg1^fl/fl^ CD23-Cre mice after 4 days activation with LPS and IL-4. Statistics were calculated using one-way ANOVA with *post hoc* Tukey’s multiple comparisons. CellTrace Violet staining indicates the magnitude of B cell proliferation.
