# Supplementary figures and images for: Brg1 Supports B Cell Proliferation and Germinal Center Formation Through Enhancer Activation
Source: Front Immunol. 2021 Sep 1;12:705848. doi: 10.3389/fimmu.2021.705848 (PMC8440861; doi:10.3389/fimmu.2021.705848)

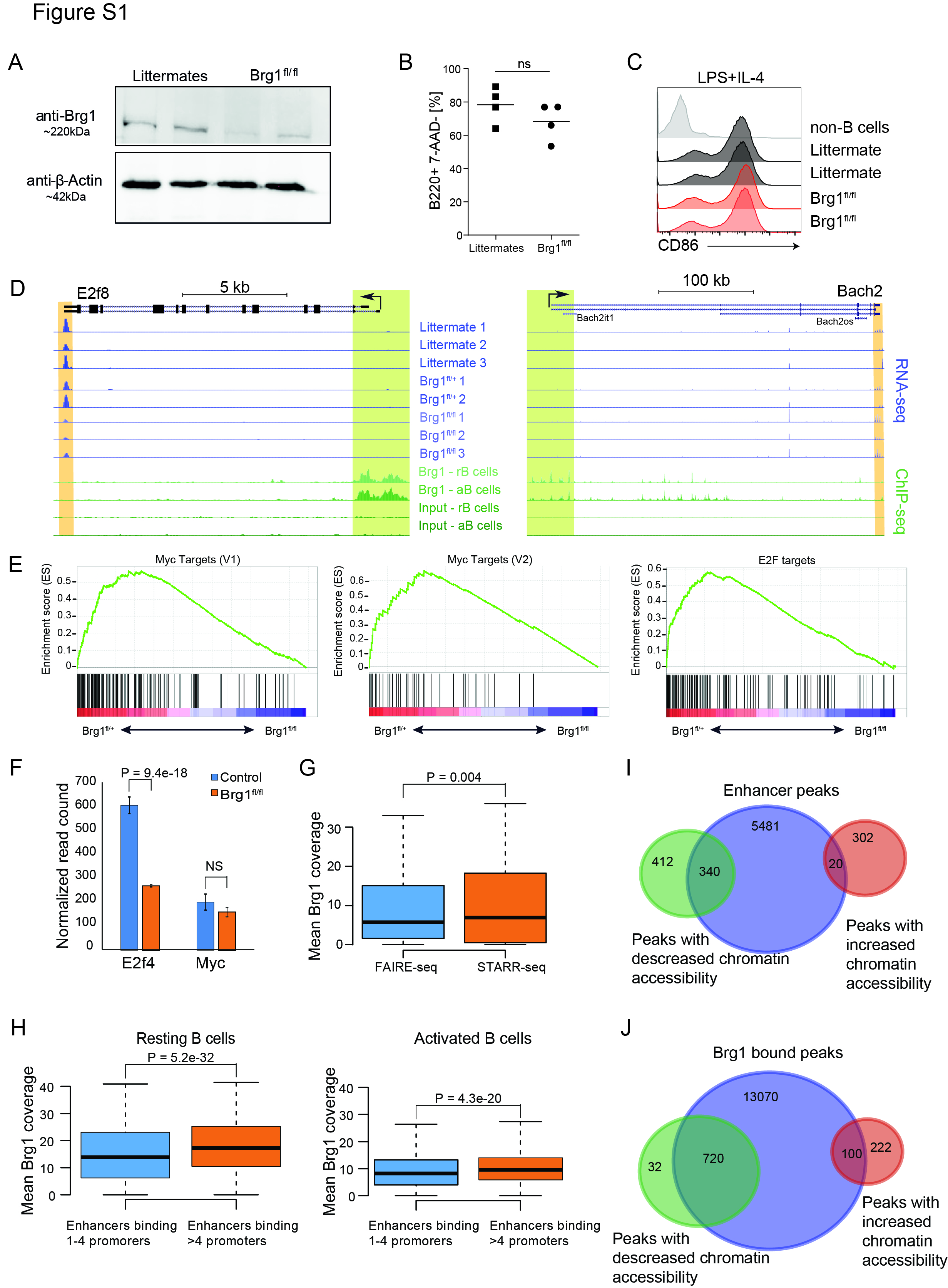

Supplement: Supplementary Figure 1 — Brg1 is required for proper expression of cell-cycle-related genes in LPS-stimulated B cells. (A) Western Blot analysis for Brg1 protein levels in splenic B cells of littermate and Brg1fl/fl CD23-Cre mice. B cells were activated for 4 days with LPS and IL-4. β-Actin serves as loading control. (B, C) Flow cytometric analysis of the live-dead marker 7-AAD as well as B cell activation marker CD86 after 18 hours of activation with LPS and IL-4. Background staining was determined from CD86-PE on B220-negative splenic cells (non-B cells). (D) UCSC genome browser tracks showing the loci of E2f8, which was significantly downregulated in CD23-Cre Brg1fl/fl B cells, and Bach2, which was significantly upregulated in CD23-Cre Brg1fl/fl cells. Tracks show the expression levels of the genes, measured by RNA-seq, in activated B cells from littermates, CD23-Cre Brg1fl/+, and Brg1fl/fl mice, and ChIP-seq signal of Brg1 in resting and activated B cells, compared to input control. Green highlight: binding of Brg1 in the promoter regions. Orange highlights: 3’ end exons covered by RNA-seq. rB cells, resting B cells. aB cells, activated B cells. (E) Gene set enrichment plots for Myc targets and E2f targets from the hallmark gene sets. (F) Expression levels of E2f4 and Myc, measured by RNA seq, in control samples, including CD23-Cre Brg1fl/fl and littermate controls. Normalization of reading counts and calculation of p value were done using Deseq2. (G) Boxplots indicating the median, quartiles, and 5th and 95th percentiles of mean Brg1 coverage across all FAIRE-seq and STARR-seq peaks in resting B cells. P values were calculated by a two-sided Wilcoxon rank-sum test. (H) Boxplots indicating the median, quartiles, and 5th and 95th percentiles of mean Brg1 coverage across all enhancers binding 1-4 promoters and enhancers binding more than 4 enhancers, in resting and activated B cells. P values were calculated by a two-sided Wilcoxon rank-sum test. (I) Venn diagram showing overlap between 5 [file Image_1.jpeg]

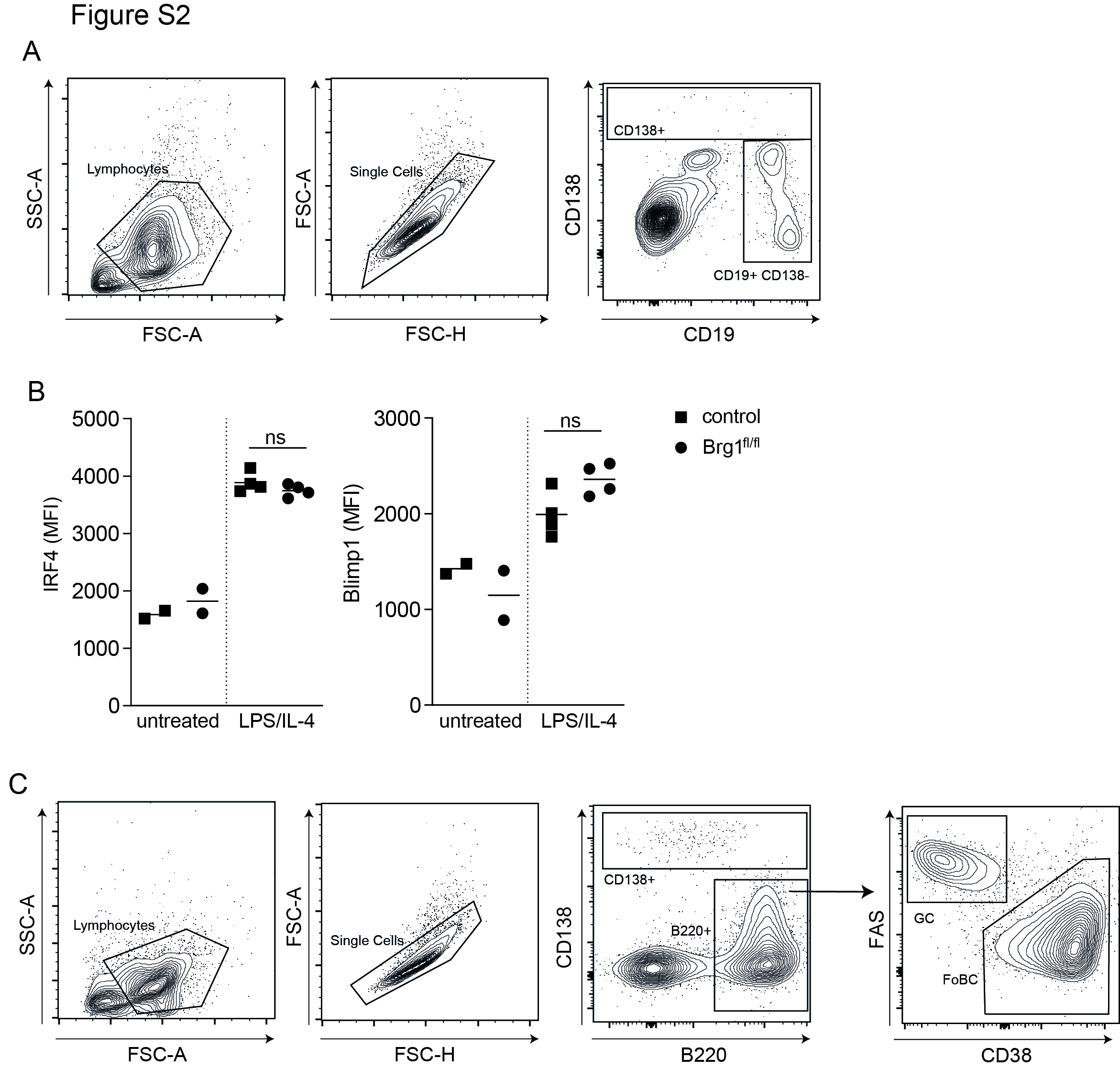

Supplement: Supplementary Figure 2 — Brg1 is not critical for the expression of Blimp-1 and IRF4 in B cells following LPS activation. (A) Gating strategy used for analysis of PCs in BM samples. (B) Analysis of IRF4 and Blimp-1 protein levels assessed by intracellular flow cytometry in CD23-Cre littermates or Brg1fl/fl splenic B cells before and after 4 days activation with LPS and IL-4. Statistics were calculated with students’ t-test. (C) Gating strategy applied to detect GC B cells in lymph nodes and PPs. [file Image_2.jpeg]

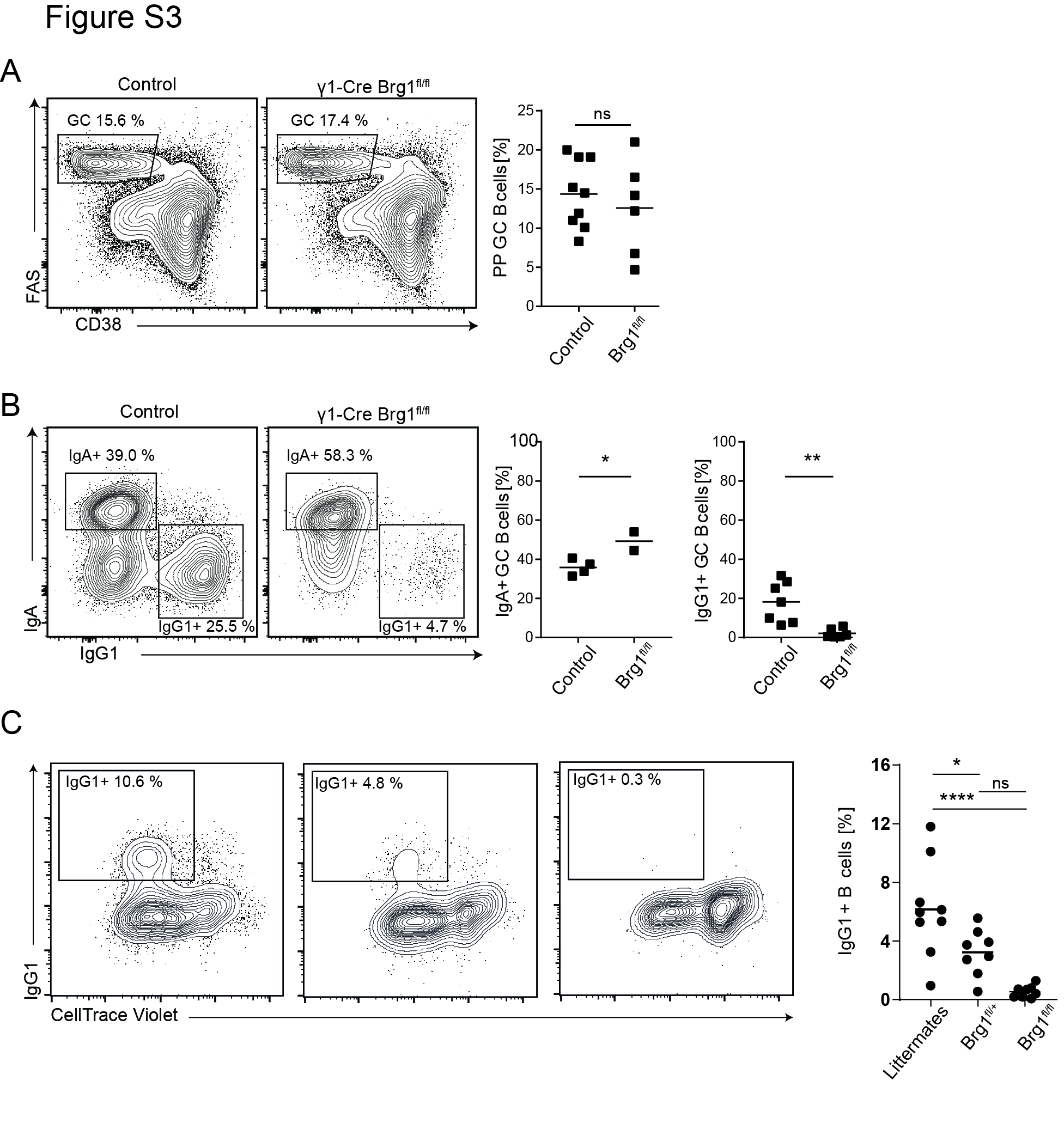

Supplement: Supplementary Figure 3 — Brg1 is required for the generation of IgG1+ germinal centers B cells in Peyer’s patches. (A) Analysis of germinal center size in PPs of γ1-Cre control (littermate or Brg1fl/+) or Brg1fl/fl mice analyzed by flow cytometry. Statistic s were calculated with students’ t-test. (B) Analysis of class-switch recombination to IgA and IgG1 in Peyer’s patches of γ1-Cre control (littermate or Brg1fl/+) or Brg1fl/fl mice analyzed by flow cytometry. Statistics were calculated with students’ t-test. (C) Analysis of class-switch recombination to IgG1 in splenic B cells derived from littermate, Brg1fl/+ or Brg1fl/fl CD23-Cre mice after 4 days activation with LPS and IL-4. Statistics were calculated using one-way ANOVA with post hoc Tukey’s multiple comparisons. CellTrace Violet staining indicates the magnitude of B cell proliferation. [file Image_3.jpeg]
